# Assessment of type 2 diabetes mellitus patients' behavioral characteristics associated with integrated treatment and prevention services in community health centers in China

**DOI:** 10.3389/fpubh.2022.1084946

**Published:** 2023-01-25

**Authors:** Ran Zhao, Nan Zhao, Sizhe Wang, Xia Zhang, Bogui Ding, Ying Li, Wenxue Miao, Lihua Pan, Hong Fan

**Affiliations:** ^1^School of Public Health, Nanjing Medical University, Nanjing, China; ^2^School of Nursing, Nanjing Medical University, Nanjing, China; ^3^Fangshan Community Health Service Center, Nanjing, China

**Keywords:** integrated treatment and prevention, behavior, cluster analysis, multiple linear regression analysis, type 2 diabetes mellitus

## Abstract

**Objective:**

The purpose of this study was to describe behavioral characteristics of type 2 diabetes mellitus (T2DM) patients, identify homogeneous clusters, and explore factors affecting behaviors associated with integrated treatment and prevention (ITP) services for T2DM in community health centers in China.

**Methods:**

A convenient sampling method was employed at a community health center between January and July 2022 in Nanjing. A total of 354 patients completed the self-reported questionnaires. After performing a Cluster Analysis to create a profile of participants' behaviors, a multiple linear regression analysis was conducted to explore the correlations between T2DM patients' characteristics and their behaviors associated with ITP services.

**Results:**

316 T2DM patients with a mean age of 72.09 years (SD = 5.96) were included. The behavior profiles of patients associated with ITP services were clustered into “Lower” (*n* = 198) and “Higher” (*n* = 118) groups, with average scores of 54.41 and 71.46, respectively. Of all the behaviors, complication examination and public health utilization scored the lowest. Health insurance, duration of disease, and treatment modality were independent predictors on the patients' behaviors associated with ITP services for T2DM.

**Conclusion:**

Patients' behaviors associated with ITP services for T2DM were moderately good (the score rate was 63.98%). Of all the behaviors, complication examination and public health service utilization scored the lowest and, as such, may warrant further research. The clustering of patients' behaviors tends to be polarization, distributed at the upper and lower ends of the behavior spectrum. It is necessary to develop and implement targeted interventions for different groups to improve T2DM patients' behaviors associated with ITP services.

## 1. Introduction

### 1.1. Prevalence of diabetes in China

The incidence and prevalence of diabetes and its complications are increasing at an alarming rate year upon year, and in turn seriously reducing patients' quality of life (1). Globally, the number of patients with diabetes is expected to rise to 642.80 million by 2030 (2). In 2019, global prevalence, number of deaths in the population, and disability-adjusted life-years (DALYs) in the population associated with diabetes were 6.18%, 1.55 and 70.88 million, respectively (3). The prevalence of diabetes in high-income countries was 8.4% in 2021. The largest prevalence of diabetes was found in upper-middle and middle-income countries, where more than 10% of the population have the condition (4, 5). The International Diabetes Federation (IDF) Diabetes ATLAS (6) indicates that China is the country with the largest number of patients with diabetes in the world. The prevalence of diabetes in China reached 11.2 percent in 2020 (7). The total diabetes-related health expenditure of China in 2021 among adults aged 20–79 years was the second highest in the world, at 165.3 billion USD. The Global Burden of Disease (GBD) (8) suggests that the annual growth rate of DALYs per 100,000 people in China was 2.27% from 1990 to 2019. Diabetes has therefore become one of the major public health problems affecting the Chinese population. Accordingly, addressing the formidable challenge posed by diabetes is a pressing concern.

### 1.2. ITP services of community health centers in China

China's health care delivery system is highly fragmented (9). In China, medical treatment services are mainly provided by general hospitals, while public health services are mainly provided by community health institutions and centers for disease prevention and control (CDC). These health institutions are often separate and unconnected. Multiple problems arise from the fragmentation of prevention and treatment of type 2 diabetes mellitus (T2DM) in China's health service system (10, 11). For example, it may prevent T2DM patients from accessing continuous health management services, such as health education, disease screening, treatment, and follow-up (12). To further promote the reform of the health system and consolidate the effects of chronic disease prevention and control, “the Medium-to-Long Term Plan of China for the Prevention and Treatment of Chronic Diseases (2017–2025)” proposes (13) to strengthen treatment and prevention collaboration, and promote the integrated development of chronic disease prevention, treatment, and management. Since 2018, the China National Health Commission has made reference to the concept of “Integrated treatment and prevention (ITP) services” in a number of documents. Although there is no specific definition of ITP services, it can be characterized, much like integrated care, as a coherent, coordinated collection of services that can be provided to patients through a variety of organizations, professionals and caregivers (14). Studies have demonstrated that combining prevention and treatment services for T2DM patients not only connects different levels of healthcare to offer patients integrated services that increase service delivery efficiency, but also has a positive impact on patients' blood glucose levels (15).

ITP services of T2DM aim to provide a full range of health services with an emphasis on integrity, coordination, and continuity (16). The quality of integrated services to some extent, can be reflected by the behavioral characteristics of patients with T2DM being treated in community health centers. To date, however, the availability of quantitative evidence on the behavioral characteristics associated with ITP services for T2DM in community health centers in China has been limited. The patients' behaviors associated with ITP services in this study were defined as the activity performances of people with T2DM from various backgrounds in terms of self-control in disease treatment and prevention. Profiling patients' behavior associated with ITP services can be helpful in terms of determining the self-management performance of people with T2DM, and can also represent how the ITP services are evolving. Understanding T2DM patients' typical behavior patterns associated with ITP services and the influencing factors can provide an accurate and objective theoretical and empirical basis for future research.

## 2. Methods

### 2.1. Study design and settings

The cross-sectional survey was conducted from January to July of 2022 in Nanjing, Jiangsu Province, China. A community health center was selected from Jiangning District as the sample institution.

### 2.2. The inclusion and exclusion criteria

#### 2.2.1. Inclusion criteria

(a) Those diagnosed with T2DM, who met the diagnostic criteria of diabetes proposed in China's Guidelines for the Prevention and Treatment of Type 2 Diabetes (2020 edition), (b) Permanent residents of the community (living in the community for more than half a year), (c) Those registered at the community health service center and a contract for diabetes management in the community, (d) Those of sound mind, with effective coordination, and communication skills, (e) Those who volunteered to participate in the research.

#### 2.2.2. Exclusion criteria

(a) Those diagnosed with other types of diabetes, such as type 1 diabetes, gestational diabetes, etc., (b) Those stricken with acute complications such as infection, ketoacidosis, hyperosmolar hyperglycemia, etc., (c) Those suffering from malignant diseases or in the advanced stage of other serious diseases, (d) Those suffering from serious complications of diabetes, such as diabetic nephropathy, fundus lesions, diabetic foot, etc., (e) Those incapable of caring for themselves due to mental illnesses or severe cognitive dysfunction, (f) Those with underlying severe hearing disorders and/or speech impairments.

### 2.3. Data collection

According to the literature, convenience sampling can be used to draw generalizations about certain sample features and is an option for researchers who are short on time, labor, etc. Therefore, this method was adopted because of its advantages in affordability, convenience, availability of research objects and applicability of research purposes. Comrey (17) contends that the sample size for general analysis is typically no <200, besides, Tinsly (18) believes that the sample size required by the research should take into account the number of variables in the study. The minimum sample size in the study of exploring influencing factors of related variables was 5–10 times the number of variables (16). As the ITP subscale has 19 items, a minimum sample size of 209 was required with a ratio of 10:1, given 10% of invalid questionnaires. A total of 354 T2DM patients (*N* = 354) were selected for this investigation by convenient sampling methods based on geographic proximity, availability and willingness to participate, and accessibility to the researchers. Due to questionnaire data loss, withdrawal, or duplicate data, 38 patients were eliminated from the investigation, and 316 questionnaires (*n* = 316) were finally included in the study, with the participants response rate of 89.27%. Data collection was carried out by trained investigators of the team according to relevant regulations and respondents were invited to complete face-to-face questionnaires containing demographic information as well as a subscale of behaviors associated with ITP services.

### 2.4. Measures

#### 2.4.1. The operational definition of ITP

The patients' behaviors associated with ITP services was mainly evaluated in seven dimensions, including dietary control, physical exercise, foot care, medication compliance, glucose and blood pressure monitoring, complication screening, and public health service utilization. The specific measurement items of each dimension were shown in the Supplementary material.

#### 2.4.2. DSKAB-SF

The brief version of Diabetes Self-management Knowledge, Attitude, and Behavior Assessment Scale (DSKAB-SF) consists of three subscales of knowledge, attitude and behavior, with a total of 42 items and a full score of 144, which can achieve the efficient evaluation of diabetic patients in daily health services (19, 20). The knowledge subscale has 22 items and 6 dimensions (basic knowledge, diet, exercise, medicine, glucose and blood pressure monitoring and hypoglycemia prevention). Each item is divided into three levels of “correct”, “unclear” and “wrong” and assigned 2, 1, and 0 points in turn, with a full score of 44. The attitude subscale consists of 5 dimensions (attitude of management, diet, exercise, medication, glucose and blood pressure monitoring) and 5 items. The options of “very important”, “important”, “general”, “unimportant” and “very unimportant” are each given 5, 4, 3, 2 or 1 points, for a total of 25. The behavioral subscale is comprised of 15 items with the items as “never”, “rarely”, “sometimes”, “often” and “always”, which are successively assigned as 1, 2, 3, 4, and 5 points, with a maximum score of 75. The 6 dimensions of the behavioral subscale are diet, exercise, prescribed medication, foot care, glucose and blood pressure monitoring and complication examination. The higher the score, the better the self-management knowledge, attitude and behavior of diabetic patients. The DSKAB-SF is rapid and thorough in its appraisal of groups or individuals' self-management of diabetes and has strong surface validity and content validity. Therefore, we adopted this brief scale in view of its advantages in good performance, high evaluation efficiency and cultural adaptability (21).

#### 2.4.3. Final questionnaire

On the basis of DSKAB-SF, we added items related to public health of diabetes with reference to the National Basic Public Health Services Standards (third edition) and the National Guidelines for the Prevention and Control of Diabetes in Primary Care (16) to develop the final questionnaire for the survey. The behavior subscale consists of 19 items and the responses range from 19 to 95 based on a 5-point Likert scale (1 = “never”, 5 = “always”), a higher result indicating healthier or more desirable behavior. The behavior subscale's Cronbach alpha in this study was 0.84. The behavior of patients associated with ITP services was graded using scoring indices (**Table 3**). Score index was calculated as follows: (*actual score*/*the highest potential score*) × 100%, a scoring of <40% is considered poor, a score of 40% to 80% was regarded moderately good, and a score of more than 80% was considered excellent (22).

### 2.5. Ethical Statement

The study was approved by the Research Ethics Committee of Nanjing Medical University, Nanjing, China and informed consent was obtained from all participants ahead of time (Approval no. 941).

### 2.6. Data analysis

IBM SPSS 26.0 (SPSS, Inc., Chicago, IL, USA) and R × 64 3.6.3 were used. The behavioral characteristics of all participants were determined applying K-means cluster analysis in accordance with the scores of each dimension in the behavior subscale. The elbow method was first used in the clustering application to determine the ideal number of clusters, followed by the NbClust function, which provides 26 different metrics, and was used to verify the optimal number of clusters. A R package fpc was then used, measuring the similarity between objects in the dataset by silhouette coefficient, to evaluate the quality of clustering. Finally, the fviz cluster function of factoextra package was used to visualize the clustering findings.

Sociodemographic factors and clinical information were the explanatory variables, and patients' behaviors associated with ITP services were the outcome variables in this study. Pearson's correlation test was used to analyze the correlation between independent variables and patients' behaviors, and multiple linear regression was conducted to present the results. Dichotomous “dummy variables” generated from the multi-categorical variables were added to the stepwise regression analysis along with the continuous variables. The Durbin-Watson statistic was computed for the independence test in the analysis, and residual histograms were shown to test for normality. In additions, we stipulated the regression models with variance inflation factors (VIF) <3 to satisfy the assumption of multicollinearity.

## 3. Results

### 3.1. Basic characteristics

The average age of the participants in this study was 72.09 ± 5.96 years, of whom 74.05% were female. The average disease course was 10.54 ± 7.32 years. 47.78% of patients were overweight. 42.09% of the patients were illiterate and 33.23% had primary education. Most patients were married or cohabiting (74.05%), unemployed (93.99%), and on a low-income (96.20%). 14.87% did not have any insurance. The majority of patients had no family history of diabetes (68.04%), but had a history of hospitalization (62.66%) and nearly half of the patients had complications of diabetes (46.52%) (Table 1).

**Table 1:** Basic characteristics of participants.

**Variables**	**Overall**	**“Low”^ 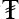 ^**	**“High”**	**t/χ^2^**	***P*-value**
**Sociodemographic data**					
Age, years (mean ± SD^*^)	72.09 ± 5.96	71.65 ± 6.02	72.84 ± 5.80	1.723	0.086
**Gender**, ***N*** **(%)**				0.185	0.693
Male	82 (25.95)	53 (26.77)	29 (24.58)		
Female	234 (74.05)	145 (73.23)	89 (75.42)		
**Education level**, ***N*** **(%)**				2.602	0.635
University and above	5(1.58)	3 (1.52)	2 (1.69)		
Senior high school/technical secondary school	15 (4.75)	10 (5.05)	5 (4.24)		
Junior high school	58 (18.35)	40 (20.20)	18 (15.25)		
Primary school	105 (33.23)	68 (34.34)	37 (31.36)		
Illiteracy	133(42.09)	77 (38.89)	56 (47.46)		
**Marriage**, ***N*** **(%)**				2.865	0.111
Single^#^	82 (25.95)	45 (22.73)	37 (31.36)		
Non-single	234 (74.05)	153 (77.27)	81 (68.64)		
**Work**, ***N*** **(%)**				2.292	0.149
Employed	19 (6.01)	15 (7.58)	4 (3.39)		
Unemployed	297 (93.99)	183 (92.42)	114 (96.61)		
**Monthly income, RMB**, ***N*** **(%)**				3.757	0.345
< 3,000	304 (96.20)	191 (96.46)	113 (95.76)		
3,000–5,000	6 (1.90)	2 (1.01)	4 (3.39)		
5,000–8,000	5 (1.58)	4 (2.02)	1 (0.85)		
≥10,000	1 (0.32)	1 (1.01)	0 (0.00)		
**Health insurance**, ***N*** **(%)**				95.198	< 0.001
State medicine	1 (0.32)	0 (0.00)	1 (0.85)		
Urban employee basic medical insurance	6 (1.90)	4 (2.02)	2 (1.70)		
Urban-Rural Resident Basic Medical Insurance	259 (81.96)	191 (96.46)	68 (57.63)		
Commercial insurance	1 (0.32)	1 (0.32)	0 (0.00)		
Other^ 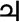 ^	2 (0.63)	0 (0.00)	2 (1.70)		
None^ 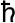 ^	47 (14.87)	2 (0.63)	45 (38.14)		
**Clinical data**					
Disease course^ 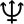 ^, years, (mean ± SD)	10.54 ± 7.32	9.77 ± 6.84	11.85 ± 7.95	2.366	0.019
**Body mass index** ^ 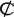 ^**, kg/m**^2^, ***N*** **(%)**				2.180	0.688
< 18.5	1 (0.32)	1 (0.32)	0 (0.00)		
18.5–24.0	100 (31.65)	64 (32.32)	36 (30.51)		
24.0–28.0	151 (47.78)	90 (45.45)	61 (51.69)		
≥28.0	64 (20.25)	43 (21.72)	21 (17.80)		
**Family history**^¤^, ***N*** **(%)**					
Yes	101 (31.96)	67 (33.84)	34 (28.81)	0.858	0.384
No	215 (68.04)	131 (66.17)	84 (71.19)		
**Hospitalization history**^ 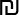 ^, ***N*** **(%)**					
Yes	198 (62.66)	123 (62.12)	75 (63.56)	0.065	0.811
No	118 (37.34)	75 (37.88)	43 (36.44)		
**Complication of diabetes**^ 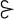 ^, ***N*** **(%)**					
Yes	147 (46.52)	92 (46.46)	55 (46.61)	0.001	1.000
NO	169 (53.48)	106 (53.54)	63 (53.39)		
**Treatment strategies**^ 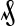 ^, ***N*** **(%)**					
Diet and exercise	1 (0.32)	1 (0.51)	0 (0.00)	7.611	0.046
Oral hypoglycemic agents	155 (49.05)	108 (54.55)	47 (39.83)		
Insulin monotherapy	122 (38.61)	68 (34.34)	54 (45.76)		
Insulin combined with oral hypoglycemic agents	38 (12.03)	21 (10.61)	17 (14.41)		

“Low”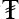: the name of cluster 1. Cluster 2 was called as “High”.

SD^*^: standard deviation.

Single^#^: including divorced or widowed.

Other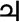: other types of insurance except state medicine, urban employee basic medical insurance (UEBMI), urban-rural resident basic medical insurance (URRBMI) and commercial insurance.

None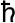: without any health insurance.

Disease course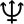 (DC): Interval between diagnosis of diabetes and completion of questionnaire.

Body mass index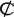 (BMI): according to National guidelines for the prevention and control of diabetes in primary care (2018), BMI (18.5–24.0) is normal, BMI (24.0–28.0) meaning overweight and BMI (≥28.0) indicates obesity.

Family history^¤^ (FH): Whether a blood relative had diabetes.

Hospitalization history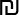 (HH): Whether the participant had experienced hospitalization due to diabetes.

Complication of diabetes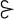 (CD): various complications of diabetes including hypertension, and other chronic diseases.

Treatment strategies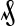 (TS): were divided into non-pharmacological diet and exercise, oral hypoglycemic agents (OHAs), mono-insulin therapy, and insulin combined with oral hypoglycemic agents (combination therapy).

### 3.2. Behavioral characteristics

**Figure 1:**
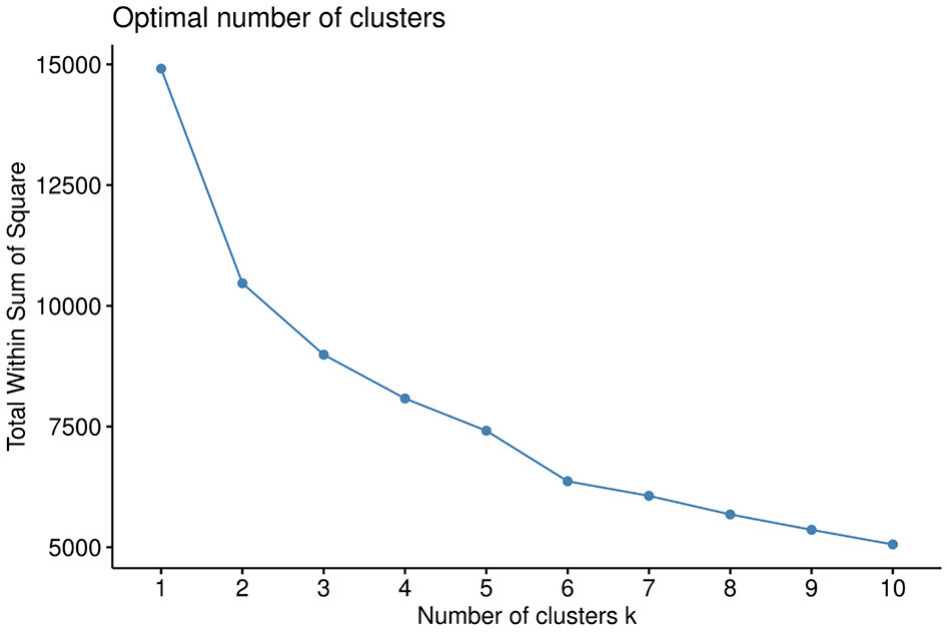
Elbow value figure of optimal number of clusters.

**Figure 2:**
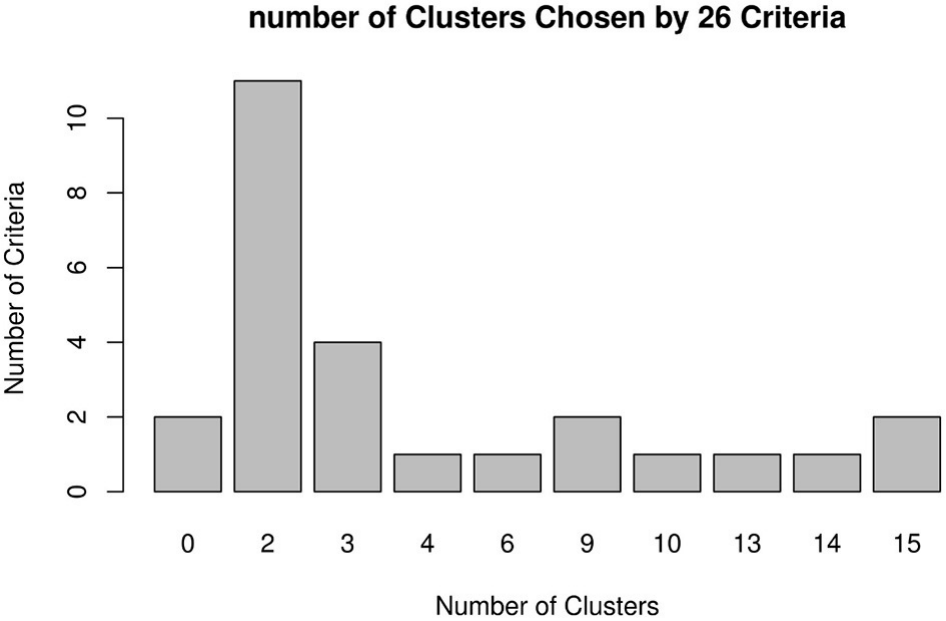
Number of clusters chosen by 26 criteria.

**Figure 3:**
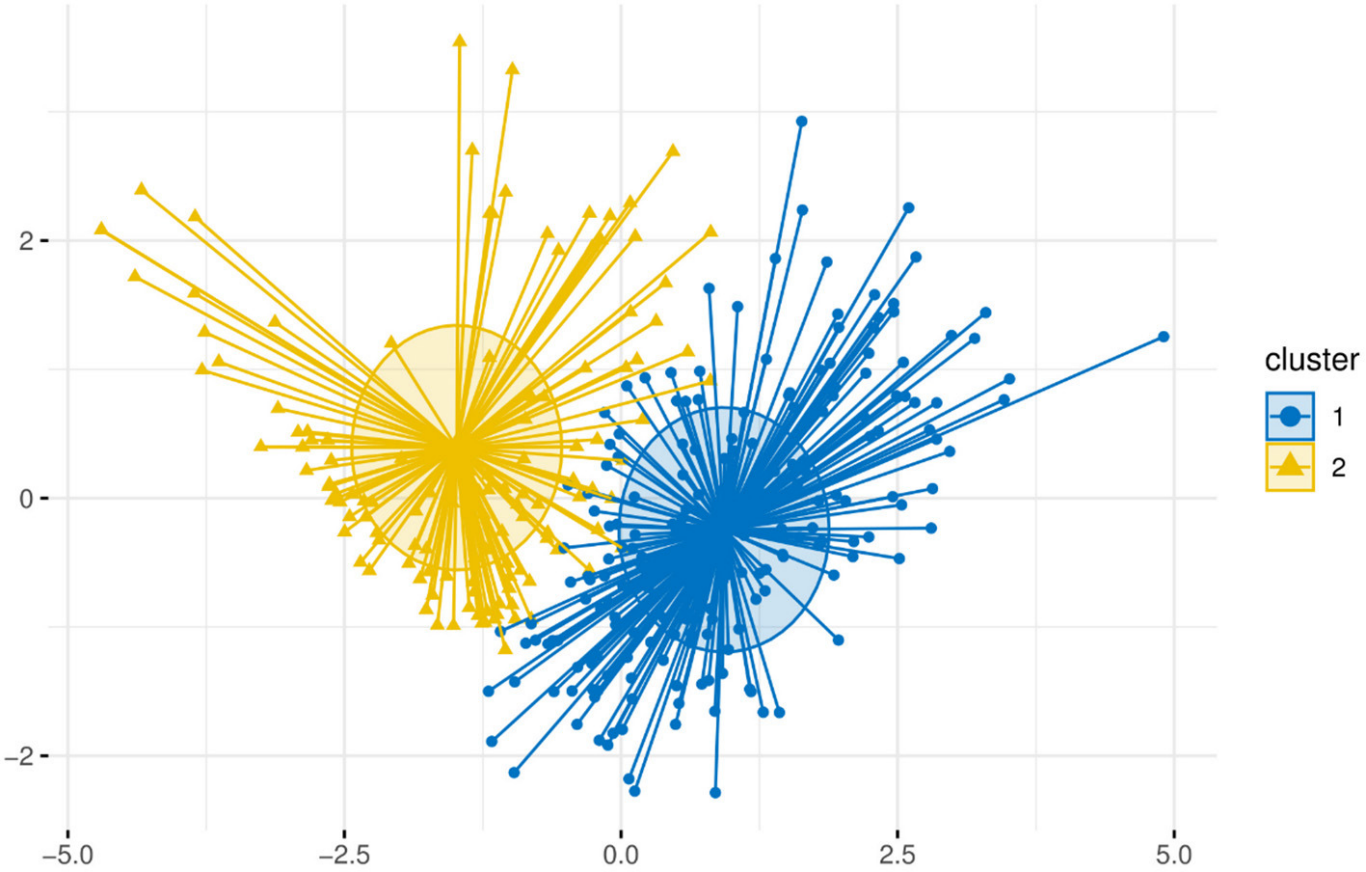
K-means clustering result. Cluster 1 (n = 198) named “Low”, presented lower scores in the behavior subscale. Cluster 2 (n = 118), assigned as “High”, scored higher on all dimensions of behaviors associated with ITP services.

In this paper, the k-mean algorithm was used for cluster analysis with patients' behavior scores of each dimension as input variables. We calculated the Within Sum of Squares (WSS) in the cluster for each k value. WSS curve was drawn according to cluster number k, and position of the inflection point (elbow) in the curve was generally regarded as an indicator of the appropriate cluster number. It can be seen the inflection point of the curve was roughly around 3 from Figure 1. Figure 2 shows that the number of indicators supporting 2 clusters is the largest. Therefore, it can be determined that the number of clusters in k-means clustering is 2. The silhouette coefficient is an evaluation index of cluster density and dispersion, and the silhouette coefficient of the clustering result in this study is 0.44 illustrating the result of sample clustering is comparatively reasonable. The scores across 7 dimensions were used as variables for cluster analysis, and the Euclidean method was used to measure the distance of the dissimilarity matrix. The obtained visual clustering results are shown in Figure 3.

The behavior subscale was used to measure the degree of patients' behavior associated with ITP services in the process of health management of T2DM, and its mean value was 60.78 (SD = 10.92, range 19–95). The patients' behavior with the highest score rate was medication compliance (score rate was 93.00%), and the patients' behavior with the lowest score rate was complication examination (score rate was 40.40%) (Table 2). The average score of patients' behavior in 198 T2DM patients in the “Low” group was 54.41 ± 6.32, among which 197 patients recorded moderately good behavioral performance, accounting for 99.49%. The mean score of patients' behavior in the “High” group was 71.46 ± 8.35. Within this group, 30 patients (33.68%) recorded excellent performance associated with ITP services, whilst 88 patients (66.32%) recorded moderately good performance (Table 3).

**Table 2:** Total and dimension scores of behavior subscale.

**Variables**	**Score**	**Score rate^Γ^ (%)**	**Rank**	**“Low”**	**“High”**	***t***	***P*-value**
Total score	60.78 ± 10.92	63.98	–	54.41 ± 6.32	71.46 ± 8.35	19.149	< 0.001
Dietary therapy	13.39 ± 2.17	89.27	2	12.97 ± 2.27	14.10 ± 1.78	4.928	< 0.001
Exercise	7.58 ± 2.18	75.80	3	6.97 ± 2.07	8.61 ± 1.95	6.973	< 0.001
Foot care	9.41 ± 3.01	62.73	5	7.79 ± 1.82	12.13 ± 2.63	15.813	< 0.001
Prescribed medication	4.65 ± 0.58	93.00	1	4.53 ± 0.66	4.85 ± 0.35	5.666	< 0.001
Glucose and blood pressure monitoring	9.65 ± 2.40	64.33	4	9.15 ± 2.23	10.50 ± 2.44	5.040	< 0.001
Complication examination	6.06 ± 3.12	40.40	7	4.81 ± 1.98	8.14 ± 3.56	9.337	< 0.001
Access to public health services	10.03 ± 3.60	50.15	6	8.20 ± 2.47	13.12 ± 3.06	14.835	< 0.001

Score rate^Γ^ = (average score/the highest possible score) × 100.

**Table 3:** Distribution characteristics of ITP behavior in two clusters.

**Variables**	**Item^ζ^**	**“Low”**			**“High”**		
		**Excellent** ^τ^	**Moderate** ^ξ^	**Poor** ^ϕ^	**Excellent**	**Moderate**	**Poor**
Total score	19	0 (0.00)	197 (99.49)	1 (0.50)	30 (25.42)	88 (74.58)	0 (0.00)
Dietary therapy	3	162 (81.82)	36 (18.18)	0 (0.00)	109 (92.37)	9 (7.63)	0 (0.00)
Exercise	2	84 (42.42)	104 (52.53)	10 (5.05)	88 (74.58)	27 (22.88)	3 (2.54)
Foot care	3	5 (2.53)	183 (92.42)	10 (5.05)	71 (60.17)	47 (39.83)	0 (0.00)
Prescribed medication	1	191 (96.46)	6 (3.03)	1 (0.50)	118 (100.00)	0 (0.00)	0 (0.00)
Glucose and blood pressure monitoring	3	24 (12.12)	160 (80.81)	14 (7.07)	34 (28.81)	80 (67.80)	4 (3.39)
Complication examination	3	2 (1.01)	65 (32.83)	131 (66.16)	22 (18.64)	79 (66.95)	17 (14.41)
Access to public health services	4	0 (0.00)	121 (61.11)	77 (38.89)	30 (25.42)	88(74.58)	0 (0.00)

Itemζ: the number of entries for behavior subscale and each dimension.

Excellentτ: good performance of integrated treatment and prevention (ITP) behavior (score index ≥ 80%).

Moderateξ: the ITP behavior of diabetics was moderately good (40%≥ score index < 80%).

Poor^ϕ^: the performance of ITP behavior in diabetic patients was poor (score index < 40%).

The mean values of each dimension of patients' behaviors associated with ITP services in the two categories are shown in Table 2. *T*-test was performed on the scores of the two subgroups, which showed that the distribution differences of each dimension between the two groups were statistically significant (*P* < 0.001).

### 3.3. Association between basic characteristics and patients' behaviors

The differences in health insurance (χ^2^ = 95.198, *P* < 0.001), disease course (*t* = 2.366, *P* < 0.05), and treatment strategies (χ^2^ = 7.611, *P* < 0.05) between cluster 1 and cluster 2 were statistically significant (Table 1). Among the “High” group, the average duration of diabetes was longer (11.85 ± 7.95). There was also a greater number of uninsured individuals (38.14%) and higher incidence of insulin use (60.17%) than among the “Low” group (Table 1).

A correlation matrix of variables in Table 4 showed significant differences in patients' behaviors among health insurance, DC, and TS (*p* < 0.05). Table 5 showed the results of the multiple linear regression analysis with insurance, DC, and TS as independent variables, and patients' behaviors as dependent variables. The final model of the stepwise regression incorporates three factors (UEBMI, URRBMI, and combination therapy). The regression equation established was Ŷ = 72.765 − 18.095X1 − 15.085X2 + 5.980X3, R^2^ = 0.294, which suggests that the independent variables included in the regression model explain 29.4% of the ITP behaviors. The regression model was statistically significant (*F* = 43.308, *p* < 0.001).

**Table 4:** A correlation matrix of variables.

	**1**	**2**	**3**	**4**	**5**	**6**	**7**	**8**	**9**	**10**	**11**	**12**	**13**	**14**
Age	1													
Gender	−0.074	1												
Education	0.161^**^	0.403^**^	1											
Marriage	0.172^**^	0.103	0.092	1										
Work	0.295^**^	0.093	0.218^**^	0.028	1									
Income	−0.333^**^	−0.197^**^	−0.274^**^	0.036	−0.252^**^	1								
Insurance	0.085	0.058	0.167^**^	0.152^**^	0.125^*^	−0.102	1							
DC	0.114^*^	0.092	0.136^*^	0.060	−0.023	−0.100	0.070	1						
BMI	0.141^*^	−0.058	0.027	0.090	0.042	−0.020	0.061	0.024	1					
FH	0.138^*^	−0.112^*^	0.039	−0.059	0.141^*^	−0.087	0.081	−0.117^*^	−0.001	1				
HH	0.040	0.024	0.039	−0.009	0.113^*^	−0.045	0.104	−0.046	0.085	0.080	1			
CD	−0.108	−0.074	−0.053	−0.128^*^	−0.129^*^	−0.012	0.140^*^	−0.073	−0.065	0.041	0.169^**^	1		
TS	−0.012	0.043	0.039	0.072	−0.041	0.083	0.021	0.324^**^	0.009	−0.020	−0.260^**^	−0.140^*^	1	
TSB^£^	0.057	0.018	0.023	0.105	0.100	−0.029	0.493^**^	0.134^*^	−0.013	0.051	0.074	0.007	0.194^**^	1

** *p* < 0.01;

* *p* < 0.05.

TSB^*$*^: total score of behavior (TSB), sum of scores for each dimension of the behavioral subscale.

**Table 5:** The association between sociodemographic variables, clinical data, and ITP behaviors.

**Variables**	**B**	**95% CI of B**	**Beta**	***t***	***p*-value**	**VIF**
Constant	72.765	(70.200, 75.331)		55.798	0.000	
UEBMI	−18.095	(−25.923, −10.268)	−0.227	−4.548	0.000	1.097
URRBMI	−15.085	(−17.863, −12.307)	−0.532	−10.684	0.000	1.096
Combination therapy	5.980	(2.843, 9.117)	0.178	3.751	0.000	1.000

## 4. Discussion

This study focused on typical patients' behaviors associated with ITP services among T2DM patients, and provides quantitative evidence available for reference to further explore the factors and mechanisms influencing ITP services in T2DM prevention and control. There are a limited number of clustering technology studies on the behavior of T2DM patients. The majority of the current studies concentrate on identifying profiles of lifestyle behaviors or self-management behaviors of T2DM patients, and exploring the influencing factors and their relationship with health outcomes (23–27). It is of great significance to identify typical behavioral patterns in T2DM patients.

The sample included in this study was primarily made up of older people with T2DM who were from low-income, low-educated, marginalized groups. As shown in Table 1, 133 (42.09%) of the participants were illiterate, 93.99% were retired, and 96.20% had a monthly income of <3,000 CNY. The majority of the participants (68.03%) were overweight or obese. The average disease course was 10.54 years, and 46.52% of those in the sample had complications. Studies have suggested that marginalized groups generally underutilize health services and that both general demographic characteristics and self-reported health status are important factors when it comes to influencing patients' health need preferences and health service utilization (28). This in turn indicates the importance of exploring influential factors on ITP behavior from the perspective of personal characteristics in order to facilitate the development of more targeted and practical ITP services.

### 4.1. Overview of patients' behaviors associated with ITP services

Patients' overall behavioral performance associated with ITP services was at a moderately good level (60.78 ± 10.92). The most frequent behaviors were, in order: medication compliance, dietary control, and exercise, according to the score rates of each behavioral dimension. This is in line with previous studies reporting that maintaining a healthy diet and high adherence to drug use are the most common behaviors among T2DM patients (29, 30). The result may be explained by the fact that relatively simple care strategies, such as diet control and medication compliance, are easier to put into practice than other measures of T2DM health management (31). Grant also reported high self-reported medication compliance among diabetic patients, positing that inefficient medications, adverse side effects, or patients' lack of belief in the ability of medications to control their illness were the main causes of low compliance in the past (32). The low-frequency behaviors were ranked in order: complications screening, public health service utilization, and foot care. Patients typically neglect daily foot care because only 5.6% of patients have diabetic feet, and some patients think that asymptomatic feet don't need to be checked frequently (33). Less than 15% of patients were checked for diabetic retinopathy and renal disease in the past year, according to the Davis Kibirige research (34), which unquestionably validates our findings about the low frequency of complication screening. Other than foot care, complication screening and public health service utilization are activities that cannot be done alone and often rely on physicians or health care institutions for their implementation. Trust and collaboration issues in the healthcare environment may affect patients' frequent or deep contact with healthcare providers (35).

The classification of patients' behaviors associated with ITP services based on data was reproducible, and the distribution made sense and was generally compatible with the state of health self-management (36, 37). Based on the behaviors scores, the patients in this study were clustered into “Low” and “High” groups. The two groups had a propensity to polarize on each behavioral dimension, as shown in Table 2. The “High” group performed superior to the “Low” group on all behavioral dimensions. This is similar to Nobel's findings, reinforcing the idea that people prefer to focus on the extremes of the range when making health-related decisions (38). The formulation of efficient and holistic preventive health interventions can be influenced by knowledge of whether and which risk factors are clustered together (39, 40). Accordingly, we believe that it is crucial to investigate socio-demographic information related to behavioral variations between the two groups is of great significance for the targeted development of health promotion strategies.

### 4.2. Complication screening

Comorbidity examination is the behavior that T2DM patients tend to overlook the most. The distribution statistics of “excellent, moderately good, and poor” in each behavioral dimension were conducted in two groups in accordance with the score index (Table 3), and it was discovered that the proportions of “poor performers” in the “Low” group (66.16%), and “moderately good performers” in the “High” group (66.95%) were the highest among both groups respectively. A combination of different elements ostensibly caused this disparity. There is proof that demographic traits and clinical indicators are positively correlated with patients' compliance with complication examination (41). Advanced age, higher socioeconomic level, better education, longer disease duration, more severe disease conditions, or well-controlled blood sugar often prompt patients to participate more actively in complication examination (42–44). The “High” group has a longer disease course which, consistent with the existing evidence, could explain the greater willingness of these patients to engage with complication examination. The treatment strategy is a further factor affecting patients' behaviors. According to the regression analysis, combination therapy had a beneficial effect on patients' behaviors since its behavior scores were 5.980 higher than those of the non-pharmacological treatment group. As stated in the literature, the advancement of T2DM symptoms frequently requires the prescription of insulin (45). On this basis, we speculated that the “High” group's increased use of insulin may be related to their inability to control their blood sugar. Uncontrolled blood sugar will motivate patients to take an active role in their health and disease prevention, but its specific mechanism of action needs to be further studied. Yi-Lin Hsieh explored the factors influencing patients' intention to receive complication examinations. They discovered that the participants' perceptions of barriers to receiving diabetes complication examinations and perceived susceptibility to such issues affected their intentions to get foot and renal screenings (46).

### 4.3. Public health service utilization and foot care

The “High” group performed significantly better than the “Low” group in terms of public health service utilization and foot care, with an average of 4.92 and 4.34 points higher, respectively. A shorter duration of diabetes in the “Low” cluster of patients in this study may be one of the reasons why fewer patients visited health centers, as health status was found to be a determinant of community health services utilization (47). It has been documented that people who report better health are less likely to use healthcare services (48). Besides, most of these patients in the “Low” cluster had better family and social support, were more likely to be married or cohabiting and in employment, and had higher instances of health insurance, which encouraged patients to have more confidence and motivation to adopt healthy maintenance behaviors and often meant that these patients were more likely to have adequate resources and support when coping with adverse events (49, 50). Research has proven that the employed and patients with health insurance tend to seek higher quality health services and technology at high-level hospitals and have less trust in community health centers (51, 52), which also gives some support to our regression results “compared with the uninsured, the UEBMI and URRBMI groups had 18.095 and 15.085 lower behavior scores, respectively.” Based on the above factors, we considered that better self-condition and adequate psychosocial coping resources increased to some extent the possibility of cross-level medical treatment and health care in the “Low” cluster of patients, which results in low utilization of primary health services (PHS). The low score for diabetic foot care in this study is consistent with previous research (53). Studies have shown that foot care is related to the duration of diabetes, medication, knowledge of diabetes and attitudes toward diabetes. The higher the education and awareness of diabetes, the better the foot care behavior (54). However, it was also reported that participants' knowledge of diabetes did not translate into action to prevent foot problems, suggesting that we need to consider specific individual characteristics and individual interactions with the environment when designing educational interventions (55).

D'Souza et al. indicated that the main predictors of self-care behavior were demographic and clinical characteristics (56). However, we found that only 29.4% of the variance in individuals' behaviors associated with ITP services was explained by demographic and clinical characteristics. We did not believe that demographic characteristics provided valid starting point for the purposes of considering patients' behaviors associated with ITP services for the treatment of T2DM in this instance. Most current interventions for chronic disease have focused on highly variable factors, such as attitudes or beliefs about illness or health. The literature suggests that patients' attitudes or beliefs about disease or health to a large extent affect the practice of receiving an examination of diabetes complications (46). A large amount of evidence has proved the effectiveness of health belief model (HBM) in predicting health behaviors (57, 58). This suggested that by bolstering health education and promoting diabetes management services, it was possible to increase patients' knowledge and self-efficiency while also increasing their intention to use ITP services. The literature states that chronic care is best delivered in collaboration, however, the fragmentation of healthcare systems may preclude this collaboration, and patients with chronic diseases frequently experience obstacles due to a lack of coordination and continuity in the healthcare system (35). As described in the Institute of Medicine Crossing the Quality Chasm report, contemporary healthcare delivery is characterized by frequent handoffs between providers, insufficient clinical follow-up, and a lack of time and resources to train patients in self-management. As a result, establishing the “partner stickiness” between patients and providers, as well as enhancing providers' patient-centered attitudes and behaviors, can be regarded as one of the effective ways to help patients improve their trust in the healthcare system and their self-care ability (35, 59). Additional goals include strengthening and broadening the primary healthcare system's health reform, enhancing the quality of primary health services, ensuring that patients receive individualized, high-quality care, defending the rights of marginalized groups, and eradicating health disparities.

## 5. Implications

This study has value both from a theoretical perspective and in terms of practical application: Firstly, it can provide a reference for the exploration and practice of a new model of ITP services for T2DM. This study identified T2DM patients' typical behaviors associated with ITP services in the community, and conduct a preliminary study of its influencing factors, which can serve as a useful guide for how to provide better community ITP services for patients. Secondly, this study makes unique contribution to the optimization of community health management strategies for T2DM, which may be used by community health institutions and CDCs to evaluate the implementation effect of ITP services and to develop personalized intervention measures for patients. Targeted optimization strategies should be adopted for the factors that lead to the formalistic and ineffective ITP services for T2DM in the community. These strategies will be crucial in promoting the modification and optimization of the structure and layout of medical resources, further improving the division of labor and cooperation mechanism of medical alliances, and improving the efficiency of diabetes prevention and control.

## 6. Limitation

The limitations of our study are briefly described below. Firstly, the patients' behaviors were roughly clustered into two categories in this study and more detailed profiles of behaviors may require more data support to achieve. Secondly, the behavioral data collected in this study are all self-reported by patients. As such, the results may be affected by social desirability bias and recall bias, and the behavior associated with ITP services may be overestimated to a certain extent. Thirdly, the study participants were recruited from a community health center in Nanjing, which may affect the representativeness of the general population. For this reason, future research should include diverse populations. Finally, the convenience sampling will cause some systematic errors, which cannot promote external validity, resulting in the findings lack of generalizability. While this method is suitable for exploratory research, future studies will try to introduce randomization to reduce the allocation and selection bias. The results of this study should be interpreted in an explorative manner due to the small sample size. In the future, a large sample size should be pursued, which should be sufficient to eliminate type I and type II statistical errors, improving the reliability of research.

## 7. Conclusion

Studies have shown that the basic demographic characteristics of patients can only explain 29.4% of the variation in patients' behavior associated with ITP services. Therefore, we believe that by strengthening the primary healthcare system, rationalizing the allocation of health resources, and improving the professional training of medical personnel starting from the policy and environment, and concentrating on the utilization of complication screening, foot care, and public health services as a breakthrough we can improve the willingness of T2DM patients to engage in ITP services. This would, in turn, result in the provision of better ITP services for T2DM patients with different backgrounds, enhance the health management of T2DM patients, and achieve better overall health outcomes.

## Data availability statement

The raw data supporting the conclusions of this article will be made available by the authors, without undue reservation.

## Author contributions

The data was collected by RZ, NZ, SW, XZ, LP, BD, YL, and WM. The field survey was coordinated with the help of BD, YL, and WM. Statistical analysis was performed by RZ and NZ. The manuscript was written by RZ. The study was conceived and manuscript was reviewed and edited by HF. All authors approved the final version of the manuscript.
