# Supplementary material for: Assessment of type 2 diabetes mellitus patients' behavioral characteristics associated with integrated treatment and prevention services in community health centers in China
Source: Front Public Health. 2023 Jan 25;10:1084946. doi: 10.3389/fpubh.2022.1084946 (PMC9905244; doi:10.3389/fpubh.2022.1084946)
Supplement: Supplementary file 1 [file Data_Sheet_1.PDF]

Questionnaire no.________

Questionnaire of integrated treatment and prevention for patients with type 2 diabetes

Dear Sir/Madam,

Hello! First of all, thank you very much for participating in this study! We are conducting this survey to learn about your understanding and behavior towards integrated treatment and prevention (ITP) for type 2 diabetes mellitus (T2DM), so that we can provide better ITP services in the future. It will take up to 15 minutes of your time. To preserve the confidentiality of the information you provide, we will use an anonymous survey. There is no correct or incorrect answer. The most important thing is to express your actual feelings. This survey is only for research purposes. Your meticulous completion of this questionnaire is the most important source of support for our research. Thank you for your patience and support! Let us all work together to ensure a healthy tomorrow!

Diabetes Health Management Research Group, Nanjing Medical University

**Part I. Basic information of patients**	
A1. Your year of birth:	________(subject to ID card registration)
A2. Your gender:	☐Men ☐Women
A3. Your height:	____ cm
A4. Your weight:	____ kg
A5. Your education level:	☐ University and above<br>☐ High school/secondary school<br>☐ Junior high school<br>☐ Primary school<br>☐ Illiteracy
A6. Your marital status:	☐ In marriage ☐ Single
A7. Your occupation:	☐ In employment ☐ Retired
A8. Your average monthly income:	☐ Less than 3000 Yuan<br>☐ 3000 (inclusive) to 5000 yuan<br>☐ 5000 (inclusive) to 8000 Yuan<br>☐ 8000 (inclusive) to 10000 yuan<br>☐ 10000 yuan or above
A9. Type of health insurance you are enrolled in (multiple options are available):	☐ State medicine<br>☐ Urban employee basic medical insurance<br>☐ Commercial insurance<br>☐ Urban-Rural Resident Basic Medical Insurance<br>☐ Other medical insurance<br>☐ No insurance
A10. Do you have a family history of diabetes?	☐Yes ☐No

A11. Do you have a history of hospitalization?	☐Yes ☐No
A12. Your diabetes course:	______years or ______months
A13. Have you had any complications due to diabetes?	☐Yes ☐No
A14. Your diabetes treatment modality:	☐ Diet and exercise<br>☐ Oral medication only<br>☐ Insulin only<br>☐ Oral medication combined with insulin<br>☐ Other

**Part II. Behavioral Scale**						
**Dimension**	**Entry**	**Always =5**	**Often =4**	**Sometimes =3**	**Rarely =2**	**Never =1**
**D1. Dietary therapy**	H1. Over the past six months, have you been taking a combination of both diet control, exercise and medication to control your blood sugar?					
	H2. Over the past six months, do you usually control the total amount of food you eat per day according to your doctor's or dietitian's instructions?					
	H3. Over the past six months, do you usually eat a low-fat, oil-free diet as recommended by your doctor or dietitian?					
**D2. Exercise**	H4. Over the past six months, have you done more than 30 minutes of moderate intensity exercise such as brisk walking and					

	jogging 5 days a week?					
	H5. Over the past six months, when you exercise, do you carry candy with you in case of hypoglycemia?					
**D3. Foot care**	H6. Have you been wearing the right size shoes and socks for six months?					
	H7. Have you checked your feet for blisters, chapped skin, chafing and other conditions during the past six months?					
	H8. Have you had your feet checked regularly in a medical institution for the past year?					
**D4. Prescribed medication**	H9. Over the past six months, have you been taking your medication on time and in accordance with your doctor's instructions?					
**D5. Glucose and blood pressure monitoring**	H10. Have you measured your blood sugar as often as your doctor ordered for the past six months?					
	H11. Do you follow your doctor's advice to check your HBA1c every 3 to 6 months?					
	H12. Have you had your blood pressure measured regularly for the past six months as required by your					

	doctor?					
**D6. Complication examination**	H13. Have you visited a medical institution regularly for blood lipid checks in the past year?					
	H14. Have you been checking your kidney function regularly in a medical institution for the past 1 year?					
	H15. Have you had your eyes checked regularly in a medical institution for the past year?					
**D7. Access to public health services**	H16. Will you receive face-to-face follow-up at least once every 3 months?					
	H17. Do you attend at least one health seminar every month?					
	H18. Have you received a health assessment for diabetic patients from a community health service in the past year?					
	H19. In the past six months, have you received personalized health guidance from community health services for diabetes patients?					
