# Supplementary material for: Assessment of type 2 diabetes mellitus patients' behavioral characteristics associated with integrated treatment and prevention services in community health centers in China
Source: Front Public Health. 2023 Jan 25;10:1084946. doi: 10.3389/fpubh.2022.1084946 (PMC9905244; doi:10.3389/fpubh.2022.1084946)
Supplement: Supplementary file 2 [file Data_Sheet_2.PDF]

STROBE Statement—Checklist of items that should be included in reports of ***cross-sectional studies***

	Item No	Recommendation
**Title and abstract**	1	(*a*) Assessment of type 2 diabetes mellitus patients' behavioral characteristics associated with integrated treatment and prevention services in community health centers in China
		(*b*) A convenient sampling method was used. After performing a Cluster Analysis to create a profile of participants' behaviors, a multiple linear regression analysis was conducted to explore the correlations between patients' behavioral characteristics and Integrated treatment and prevention (ITP) services. 316 type 2 diabetes mellitus (T2DM) patients with a mean age of 72.09 years (SD=5.96) were included. The behavior profiles of patients associated with ITP services were clustered into "Lower" (n=198) and "Higher" (n=118) groups, with average scores of 54.41 and 71.46, respectively. Of all the behaviors, complication examination and public health utilization scored the lowest. Health insurance, duration of disease, and treatment modality were independent predictors on the patients' behaviors associated with ITP services for T2DM. The clustering of patients' behaviors tends to be polarization, distributed at the upper and lower ends of the behavior spectrum. It is necessary to develop and implement targeted interventions for different groups to improve behaviors associated with ITP services.
**Introduction**		
Background/rationale	2	ITP services of T2DM aim to provide a full range of health services with an emphasis on integrity, coordination, and continuity. The quality of integrated services to some extent, can be reflected by the behavioral characteristics of patients with T2DM being treated in community health centers. To date, however, the availability of quantitative evidence on the behavioral characteristics associated with ITP services for T2DM in community health centers in China has been limited.
Objectives	3	The purpose of this study was to describe behavioral characteristics of T2DM patients, identify homogeneous clusters, and explore factors affecting ITP services for T2DM in community health centers in China.
**Methods**		
Study design	4	A cross-sectional survey
Setting	5	The survey was conducted from January to July of 2022 in Nanjing, Jiangsu Province, China. A community health center was selected from Jiangning District as the sample institution, and the total sample size of the study design was confirmed to be 354 participants through convenience sampling.<br>Data collection was carried out by trained investigators of the team according to relevant regulations and respondents were invited to complete face-to-face questionnaires containing demographic information as well as a subscale of ITP behaviors.
Participants	6	**Inclusion criteria:** a) those diagnosed with T2DM, who met the diagnostic criteria of diabetes proposed in China's Guidelines for the Prevention and Treatment of Type 2 Diabetes (2020 edition), b) permanent residents of the community (living in the community for more than half a year), c) those registered at the community health service center and a contract for diabetes management in the community, d) those of sound mind, with effective coordination, and communication skills, e) those who volunteered to participate in the research.

		**Exclusion criteria:** a) those diagnosed with other types of diabetes, such as type 1 diabetes, gestational diabetes, etc., b) those stricken with acute complications such as infection, ketoacidosis, hyperosmolar hyperglycemia, etc., c) those suffering from malignant diseases or in the advanced stage of other serious diseases, d) those suffering from serious complications of diabetes, such as diabetic nephropathy, fundus lesions, diabetic foot, etc., e) those incapable of caring for themselves due to mental illnesses or severe cognitive dysfunction, f) those with underlying severe hearing disorders and/or speech impairments.<br>**Methods of selection:** convenience sampling
Variables	7	Sociodemographic factors and clinical information were the explanatory variables, and patients' behaviors associated with ITP services were the outcome variables in this study.
Data sources/ measurement	8*	The patients' behaviors associated with ITP services were mainly evaluated in seven dimensions, including dietary control, physical exercise, foot care, medication compliance, blood glucose and blood pressure monitoring, complication screening, and public health service utilization (see Supplementary).<br>On the basis of the brief version of Diabetes Self-management Knowledge, Attitude, and Behavior Assessment Scale (DSKAB-SF), we added items related to public health of diabetes with reference to the National Basic Public Health Services Standards (third edition) and the National Guidelines for the Prevention and Control of Diabetes in Primary Care (2018) to develop the final questionnaire for the survey. The behavior subscale consists of 19 items and the responses range from 19 to 95 based on a 5-point Likert scale (1= "never," 5= "always"), a higher result indicating healthier or more desirable behavior. The original simplified scale has good reliability and validity and can quickly and comprehensively evaluate the self-management of patient groups or individuals. The behavior subscale's Cronbach alpha in this study was 0.84. The behavior of patients associated with ITP services was graded using scoring indices (Table 3). Score index was calculated as follows: actual score/the highest potential score)×100%, a scoring of less than 40% is considered poor, a score of 40% to 80% was regarded moderately good, and a score of more than 80% was considered excellent.
Bias	9	The behavioral data collected in this study are all self-reported by patients. As such, the results may be affected by social desirability bias, and the behavior associated with ITP services may be overestimated to a certain extent.<br>As a type of non-probability sampling, convenience sampling will produce some systematic errors.
Study size	10	The minimum sample size in the study of exploring influencing factors of related variables was 5-10 times the number of variables. As the ITP subscale has 19 items, a minimum sample size of 209 was required with a ratio of 10:1, given 10% of invalid questionnaires.
Quantitative variables	11	Quantitative variables of demographic information were analyzed by means of descriptive statistics;<br>Groupings were chosen by K-means clustering.
Statistical methods	12	(*a*) K-means clustering, descriptive statistics, Pearson's correlation test, multiple linear regression
		(*b*) Pearson's correlation test was used to analyze the correlation between independent variables and patients' behaviors, and multiple linear regression was conducted to present the results. Dichotomous "dummy variables" generated from the multi-

		categorical variables were added to the stepwise regression analysis along with the continuous variables.
		(*c*) Eliminating invalid questionnaires
**Results**		
Participants	13*	A total of 354 T2DM patients (N=354) were selected for this investigation by convenient sampling methods based on geographic proximity, availability and willingness to participate, and accessibility to the researchers. Due to questionnaire data loss, withdrawal, or duplicate data, 38 patients were eliminated from the investigation, and 316 questionnaires (n=316) were finally included in the study, with the participants response rate of 89.27%.
Descriptive data	14*	The average age of the participants in this study was 72.09±5.96 years, of whom 74.05% were female. The average disease course was 10.54±7.32 years. 47.78% of patients were overweight. 42.09% of the patients were illiterate and 33.23% had primary education. Most patients were married or cohabiting (74.05%), unemployed (93.99%), and on a low-income (96.20%). 14.87% did not have any insurance. The majority of patients had no family history of diabetes (68.04%), but had a history of hospitalization (62.66%) and nearly half of the patients had complications of diabetes (46.52%) (Table 1).
Outcome data	15*	Not Applicable
Main results	16	(*a*) In this paper, the number of clusters in k-means clustering is 2. The silhouette coefficient is 0.44 illustrating the result of sample clustering is comparatively reasonable. The scores across 7 dimensions were used as variables for cluster analysis, and the euclidean method was used to measure the distance of the dissimilarity matrix. The obtained visual clustering results are shown in Figure 3.
		(*b*) The mean value of patients' behavior associated with ITP services was 60.78 (SD=10.92, range 19-95). The patients' behavior with the highest score rate was medication compliance (score rate was 93.00%), and the patients' behavior with the lowest score rate was complication examination (score rate was 40.40%) (Table 2). The average score of patients' behavior in 198 T2DM patients in the "Low" group was 54.41±6.32, among which 197 patients recorded moderately good behavioral performance, accounting for 99.49%. The mean score of patients' behavior in the "High" group was 71.46±8.35. Within this group, 30 patients (33.68%) recorded excellent performance associated with ITP services, whilst 88 patients (66.32%) recorded moderately good performance (Table 3). The mean values of each dimension of patients' behaviors associated with ITP services in the two categories are shown in Table 2. T-test was performed on the scores of the two subgroups, which showed that the distribution differences of each dimension between the two groups were statistically significant (P<0.001).
		(*c*) The differences in health insurance ($\chi2$=95.198, P<0.001), disease course (t=2.366, P<0.05), and treatment strategies ($\chi2$=7.611, P<0.05) between cluster 1 and cluster 2 were statistically significant (Table 1). Among the "High" group, the average duration of diabetes was longer (11.85±7.95). There was also a greater number of uninsured individuals (38.14%) and higher incidence of insulin use (60.17%) than among the "Low" group (Table 1).
Other analyses	17	A correlation matrix of variables in Table 4 showed significant differences in patients' behaviors among health insurance, DC, and TS (p<0.05). Table 5 showed the results of the multiple linear regression analysis with insurance, DC, and TS as independent variables, and patients' behaviors as dependent variables. The final model of the

		stepwise regression incorporates three factors (UEBMI, URRBMI, and combination therapy). The regression equation established was $\hat{Y}$=72.765-18.095X1-15.085X2=5.980X3, R2=0.294, which suggests that the independent variables included in the regression model explain 29.4% of the ITP behaviors. The regression model was statistically significant (F=43.308, p<0.001).
**Discussion**		
Key results	18	Studies have shown that the basic demographic characteristics of patients can only explain 29.4% of the variation in patients' behavior associated with ITP services. Therefore, we believe that by strengthening the primary healthcare system, rationalizing the allocation of health resources, and improving the professional training of medical personnel starting from the policy and environment, and concentrating on the utilization of complication screening, foot care, and public health services as a breakthrough we can improve the willingness of T2DM patients to engage in ITP services. This would, in turn, result in the provision of better ITP services for T2DM patients with different backgrounds, enhance the health management of T2DM patients, and achieve better overall health outcomes.
Limitations	19	The limitations of our study are briefly described below. Firstly, the patients' behaviors were roughly clustered into two categories in this study and more detailed profiles of behaviors may require more data support to achieve. Secondly, the behavioral data collected in this study are all self-reported by patients. As such, the results may be affected by social desirability bias and recall bias, and the behavior associated with ITP services may be overestimated to a certain extent. Thirdly, the study participants were recruited from a community health center in Nanjing, which may affect the representativeness of the general population. For this reason, future research should include diverse populations. Finally, the convenience sampling will cause some systematic errors, which cannot promote external validity, resulting in the findings lack of generalizability. While this method is suitable for exploratory research, future studies will try to introduce randomization to reduce the allocation and selection bias. The results of this study should be interpreted in an explorative manner due to the small sample size. In the future, a large sample size should be pursued, which should be sufficient to eliminate type Ⅰ and type Ⅱ statistical errors, improving the reliability of research.
Interpretation	20	This study has value both from a theoretical perspective and in terms of practical application: Firstly, it can provide a reference for the exploration and practice of a new model of ITP services for T2DM. This study identified T2DM patients' typical behaviors associated with ITP services in the community, and conduct a preliminary study of its influencing factors, which can serve as a useful guide for how to provide better community ITP services for patients. Secondly, this study makes unique contribution to the optimization of community health management strategies for T2DM, which may be used by community health institutions and CDCs to evaluate the implementation effect of ITP services and to develop personalized intervention measures for patients. Targeted optimization strategies should be adopted for the factors that lead to the formalistic and ineffective ITP services for T2DM in the community. These strategies will be crucial in promoting the modification and optimization of the structure and layout of medical resources, further improving the division of labor and cooperation mechanism of medical alliances, and improving the efficiency of diabetes prevention and control.
Generalisability	21	Convenience sampling does not promote external validity, so the results of this study

		are not enough to generalize to the general population of patients with T2DM.
**Other information**		
Funding	22	This research was supported by Priority Academic Program Development of Jiangsu Higher Education Institutions (PAPD). The funder had no role in the design and conduct of the study, data collection, analysis, and interpretation; writing of the manuscript; or the decision to submit the article for publication.
